# Human Tears Reveal Insights into Corneal Neovascularization

**DOI:** 10.1371/journal.pone.0036451

**Published:** 2012-05-09

**Authors:** Nadia Zakaria, Sigi Van Grasdorff, Kristien Wouters, Jos Rozema, Carina Koppen, Eva Lion, Nathalie Cools, Zwi Berneman, Marie-José Tassignon

**Affiliations:** 1 Department of Ophthalmology, Antwerp University Hospital, Antwerp, Belgium; 2 Center for Cell Therapy and Regenerative Medicine, Antwerp University Hospital, Antwerp, Belgium; 3 Department of Statistics, Antwerp University Hospital, Antwerp, Belgium; 4 Laboratory of Experimental Heamatology, Vaccine and Infectious Disease Institute (Vaxinfectio) University of Antwerp, Antwerp, Belgium; Ottawa Hospital Research Institute, Canada

## Abstract

Corneal neovascularization results from the encroachment of blood vessels from the surrounding conjunctiva onto the normally avascular cornea. The aim of this study is to identify factors in human tears that are involved in development and/or maintenance of corneal neovascularization in humans. This could allow development of diagnostic tools for monitoring corneal neovascularization and combination monoclonal antibody therapies for its treatment. In an observational case-control study we enrolled a total of 12 patients with corneal neovascularization and 10 healthy volunteers. Basal tears along with reflex tears from the inferior fornix, superior fornix and using a corneal bath were collected along with blood serum samples. From all patients, ocular surface photographs were taken. Concentrations of the pro-angiogenic cytokines interleukin (IL)-6, IL-8, Vascular Endothelial Growth Factor (VEGF), Monocyte Chemoattractant Protein 1 (MCP-1) and Fas Ligand (FasL) were determined in blood and tear samples using a flow cytometric multiplex assay. Our results show that the concentration of pro-angiogenic cytokines in human tears are significantly higher compared to their concentrations in serum, with highest levels found in basal tears. Interestingly, we could detect a significantly higher concentration of IL- 6, IL-8 and VEGF in localized corneal tears of patients with neovascularized corneas when compared to the control group. This is the first study of its kind demonstrating a significant difference of defined factors in tears from patients with neovascularized corneas as compared to healthy controls. These results provide the basis for future research using animal models to further substantiate the role of these cytokines in the establishment and maintenance of corneal neovascularization.

## Introduction

 The cornea’s main function is to allow light into the eye whilst performing the majority of the refraction. Since the cornea needs to be avascular in order to carry out its function, it receives its nutrition through the tear film and aqueous humor [1]. Tears play an essential role in maintaining homeostasis of the ocular surface. Therefore changes in the delicate equilibrium of tear cytokines may manifest themselves as various pathophysiological conditions.

Angiogenesis is the sprouting of new blood vessels from pre-existing ones and plays a crucial role in physiological and pathological processes. In response to hypoxia, tissues release pro-angiogenic growth factors and cytokines which then diffuse to nearby endothelial cells. Receptors stimulated on the endothelial cells further trigger production of proteases that result in extracellular matrix degradation [2]. Once activated, the endothelial cells proliferate and migrate in the direction of the growth factors. Finally the endothelial cells halt proliferation and begin their organization into tubules [3]. In order to stabilize the newly formed blood vessels, pericytes and smooth muscle cells surround them to establish mature blood vessels [2], [4]–[7]. There are a number of cytokines, chemokines and growth factors that could possibly influence angiogenesis in the cornea. In this study we will focus on interleukin (IL)-6, IL-8, Monocyte Chemoattractant Protein 1 (MCP-1), Vascular Endothelial Growth Factor (VEGF) and soluble Fas Ligand (sFasL) and their interactions in the tears of patients with neovascularized corneas. These cytokines were chosen since IL-6, IL-8, MCP-1 have been measured in tears of normal subjects whereas, VEGF is a known factor mediating corneal neovascularization. sFasL is also a known factor present at the ocular surface, and therefore chosen as part of this study.

Treatments for vascularized corneas have included a non randomized observational case series using topical monoclonal anti-VEGF antibody [8]. This showed reduction in corneal neovascularization in the short term results, however the high risk of side effects deemed its continuation unsafe. Here, we want to investigate VEGF and its correlation with other angiogenic cytokines (IL-6, IL-8, MCP-1 and FasL) at the ocular surface, in order to deduce which other anti-angiogenic analytes could augment the role of anti-VEGF therapies.

The aim of this study is to identify factors in human tears that may be involved in development and/or maintenance of corneal neovascularization in humans. Since tears are a dynamic reflection of the factors present at the ocular surface, this is an ideal substrate to investigate.

## Methods

Tears will be analyzed for multiple angiogenic cytokines (VEGF, FasL, MCP-1, IL-6 and IL-8) using a multiplex cytometric bead array. Different tear collection points will be evaluated to determine the source of secretion of these analytes, i.e the lacrimal gland, conjunctiva or cornea. Using this data, we will also be able to determine the optimal site and method of tear collection for analysis of analytes present in vascularized corneas. In parallel, tears from both patient and control groups will be analyzed for concentrations of these analytes and will be compared to the relative concentrations of analytes found in serum. Concomitant to the analysis of the tear film, we will measure the percentage area of corneal vascular encroachment using digital ocular photograph analyses to see if these events are correlated.

### Patients and Healthy Controls

A total of 12 patients were enrolled in this study: 6 males and 6 females; 49.7±22.3 years (mean ± standard deviation), range 27–96 years. They were screened by slit lamp examination and each prospective patient had been diagnosed with corneal neovascularization (Table 1). The patients did not to instill any ocular medication on the day of tear collection. All tear samples were collected at 14 h00 in order to avoid diurnal variation. Ten healthy volunteers constituted the control group: 5 males and 5 females; 43.9±13.9 years, range 27–59 years. These subjects were healthy, not pregnant or taking any medication and had no history of ophthalmic disease. At the time of tear collection, they did not have any ocular symptoms and were not contact lens users. Written informed consent was obtained from all subjects after they were informed of the nature and possible risks of the study. The study was approved by the Antwerp University Hospital Ethics Committee (Belgian Registration number: B30020095563) and followed the Tenets of the declaration of Helsinki.

**Table 1:** List of patients with corneal neovascularization and the corresponding diagnosis.

Subject no.	Affected eye	Diagnosis
1	ODS	LSCD secondary to aniridia
2	OD	LSCD secondary to bacterial keratitis
3	ODS	LSCD secondary to chemical burn
4	OS	LSCD secondary to bacterial keratitis
5	OS	Vascularized corneal ulcer
6	OD	Iaotrogenic LSCD
7	ODS	LSCD secondary to chemical burn
8	OS	LSCD secondary to chemical burn
9	OD	Vascularized corneal ulcer
10	OS	LSCD secondary to chemical burn
11	OD	LSCD secondary to chemical burn
12	ODS	LSCD secondary to aniridia

LSCD =  Limbal stem cell deficiency, OD = right eye, OS = left eye, ODS = both eyes.

### Tear Sample Collection

**Figure 1:**
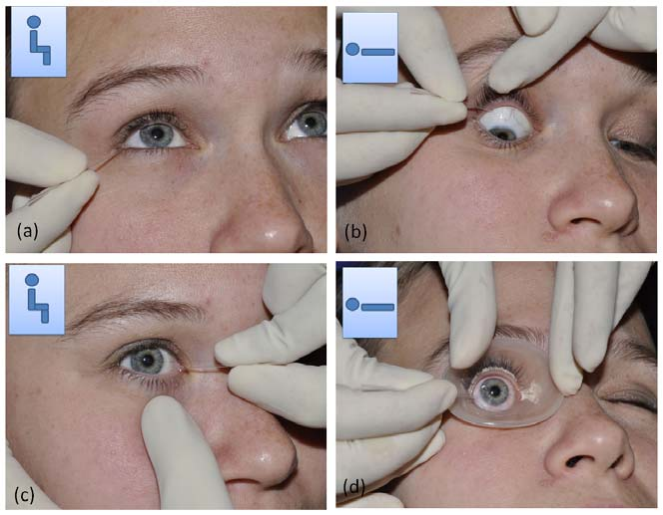
Methods of tear collection using glass micropipettes. Inset indicates the position of the subject (sitting or supine) during collection. (a) Basal tear collection, (b) Reflex lying (RL) tears are collected from the tears pooling in the superior fornix near the lateral canthus as soon as the tears exit the lacrimal gland onto the ocular surface. (c) Reflex Sitting (RS) tear collection performed from the lower fornix near the inner canthus, just before the tears leave the ocular surface through the nasolacrimal duct. (d) Corneal epithelial secretions are collected after instilling local anesthetic, using a corneal bath: 100 µl of normal saline is added to the silicone corneal bath and collected immediately.

Figure 1 shows how the different tears were collected. First the basal tears (BT) were collected from the right eye then the left eye (Figure 1a). This was done using glass capillary micropipettes (Drummond Scientific, Belgium). 10 µl of basal tears were collected from the external canthus of the inferior fornix, without eliciting reflex tearing by coming into contact with the conjunctiva. Next, the subject was made to lie supine and a bright light was shone into the eye to elicit reflex tearing (RL; Figure 1b). 35 µl of reflex tears were collected from the superior fornix by gently pulling back the upper eye lid and collecting the tears that pooled within the fornix at the exterior canthus near the anatomical opening of the main lacrimal gland. After collecting Reflex tears from both eyes while the patient was Lying (RL), the subject was made to sit upright and reflex tears from both eyes were collected from the inferior fornix near the inner canthus, i.e. Reflex Sitting tears (RS; Figure 1c). Finally the patient was made to lie supine once again and one drop of topical oxybuprocaine hydrochloride 0.4% was instilled in each eye (Figure 1d). A sterile, silicone rubber cornea bath was applied to the cornea and 100 µl of normal saline was added to the bath. The 50 µl of the diluted tears were immediately picked up using the glass micropipette. All tear samples were collected by the same clinician and dispensed into cryovials and stored immediately at −80°C. For analysis, the samples were divided into 3 groups: 1. Normal Control (NC) group, i.e. all eyes from the control group, 2. Patient Control (PC) group, i.e. the normal eyes from the patient group and 3. Patient Affected (PA) group, i.e. the neovascularized eyes from the patient group. Of the 198 samples collected, 20 (10%) consisted of volumes below the minimum cut off (5 µl) and therefore excluded from further analysis.

### Blood Sample Collection

Following tear sample collection, 10 ml of venous blood was drawn from each subject. The whole blood was allowed to clot for 30 mins at room temperature and then centrifuged at 2000 rpm for 15 mins. The serum was then separated and stored at −80°C.

### Image Analysis for Corneal Angiogenesis

**Figure 2:**
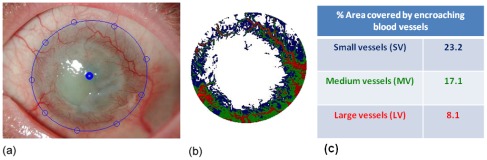
A representative example of an ocular surface photograph within the image processing software. (a) The solid blue line demarcates the limbus from the vascular cornea. In (b) the output image is illustrated with the large vessels in red, medium sized vessels in green and small vessels in blue. In (c) the calculated percentage area covered by each vessel type is depicted.

For each eye a set of digital color images were taken using a Nikon FS-3V slit lamp camera, making sure the entire limbus was visible (Figure 2). These images were analyzed using a homemade software program based on the Matlab program (version 6, The MathWorks, MA, USA). For analysis an oval shape is placed over the limbus by manually selecting a number of points on the limbal edge. Next, the specular reflections within this selected region are detected using a threshold algorithm in order to exclude them from further processing. In the remaining regions, the grey values of the green channel are subtracted from those of the red channel, giving maximal contrast to the red blood vessels. The analysis then consists of defining the grey value threshold of respectively small, medium and large blood vessels which are represented in false colors. Once the color distribution in the false color image corresponds with the blood vessel distribution in the original picture, the procedure is completed. The output is then the percentage of the limbal area covered by blood vessels of the three different diameters. All analyses were performed by the same investigator who was not made aware of the patients’ cytokine values, avoiding any bias in the execution of the procedure.

### Analysis of Cytokine Concentration

The concentration of 5 angiogenic molecules (IL-6, IL-8, MCP-1, VEGF and sFasL) was determined by a multiplex cytometric bead assay (CBA; BD Biosciences Pharmingen, San Diego, CA) according to the manufacturer’s instructions. Flow cytometric detection of cytokines was assessed at once in tear and serum samples. Standard curves of each analyte reached a top concentration of 10000 pg/mL. All samples were acquired on a FACSarray cytometer (BD Bioschiences, San Jose, CA, USA) and data were analyzed using FCAP Array v1.0.1 for windows (Soft Flow Inc., USA).

### Statistical Analysis

Serum levels in control subjects and patients were compared with an unpaired t-test. Mixed effects models were used to compare the cytokine levels in the tears. A mixed effects model accounts for the fact that measurements obtained from the same subject tend to be correlated. With this model cytokine levels from patient control (PC), patient affected (PA) and normal control (NC) eyes can be compared, as well as levels obtained under different conditions (basal, reflex lying, reflex sitting). Similarly, a mixed effects logistic regression model was used for the dichotomized outcomes. A false discovery rate (FDR) correction was made to the *p*-values to adjust for multiple testing. For the patient affected eyes, correlations between the area of vascularization and the cytokine levels were calculated by a linear regression model, from which the correlation and 95% confidence intervals were obtained. All analyses were done in SAS 9.2 (SAS Institute Inc, Cary, NC, USA).

## Results

### Tear Cytokines in Control Subjects

**Figure 3:**
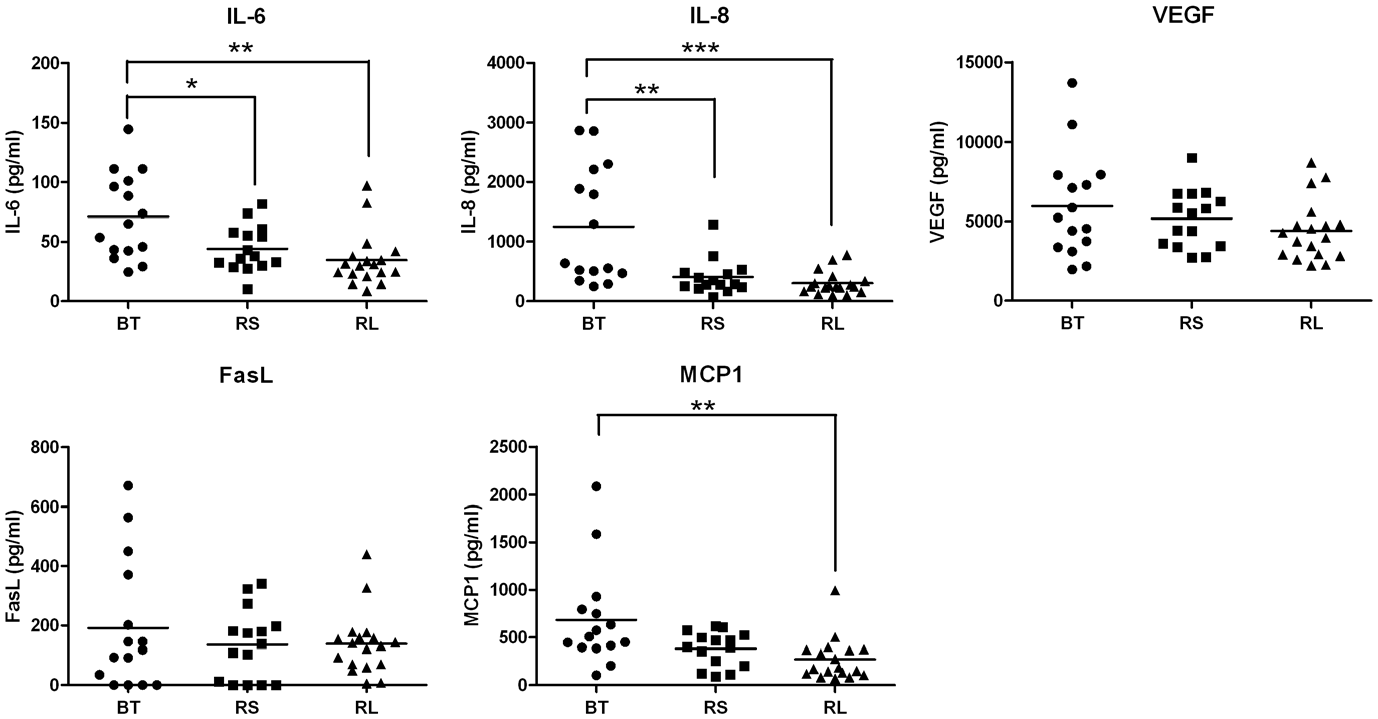
Cytokine concentrations of Basal Tears (BT), Reflex tears obtained in Sitting position (RS) and Reflex tears collected with the subject Lying (RL) in Normal Control (NC) eyes. Tears of 10 control subjects were analysed for cytokine secretion using a flow cytometry based assay. Bars indicate mean concentrations and differences are significant if *p<0.05 **p<0.001, ***p<0.0001.

Normal subjects (NC group) showed variation in tear cytokine levels form the different collection points (Figure 3). Highest levels were seen in the BT samples followed by the RS and RL. Of the 5 analytes measured, IL-8, IL-6 and MCP-1 showed significant differences between the collection points (Figure 3, *p*<0.05). There were significantly lower levels of IL-6 detected in RL compared to BT, (*p* = 0.0023) and RS compared to BT (*p* = 0.0232). Similarly, IL-8 also showed significantly lower levels in RL compared to BT (*p* = 0.0012), and demonstrated significantly lower levels in RS versus BT (*p* = 0.0028). Further, MCP-1 showed a significant reduction in RL compared to BT (*p* = 0.0027). Corneal samples were not compared because the tears were diluted during collection, unlike BT, RS and RL tear samples.

### Cytokines in Basal Tears and Serum

**Figure 4:**
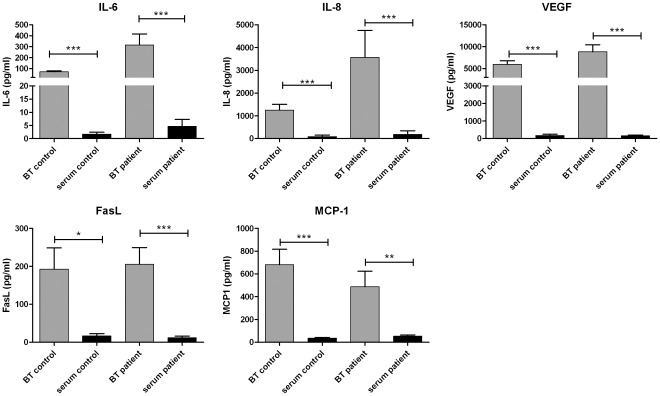
Concentration of cytokines in basal tears (BT) and blood serum of patients with neovascularized cornea’s and healthy controls. Concentrations in tear samples (grey) and serum samples (black) are depicted as mean values ± SEM for the patient group (PA+PC, n = 23) and control group (NC, n = 15). Differences are significant if ***p<0.0001, **p<0.001, *p<0.05.

The cytokine profile of BT and serum was compared for the patient (PA+PC; n = 23) and control (NC; n = 15) groups (Figure 4). Concentrations of all cytokines tested were significantly lower in serum (*p*<0.05) than those observed in BT for both the patient group as well as the control group. It is important to note that the serum samples (n = 20, 10 patient and 10 control) were not diluted so that the low values observed could not be ascribed to high sample dilution. These results clearly show large statistically significant differences between the cytokine levels detected in basal tears compared to the levels present in the serum for all 5 cytokines.

### Cytokine Profile in Basal and Reflex Tears

**Figure 5:**
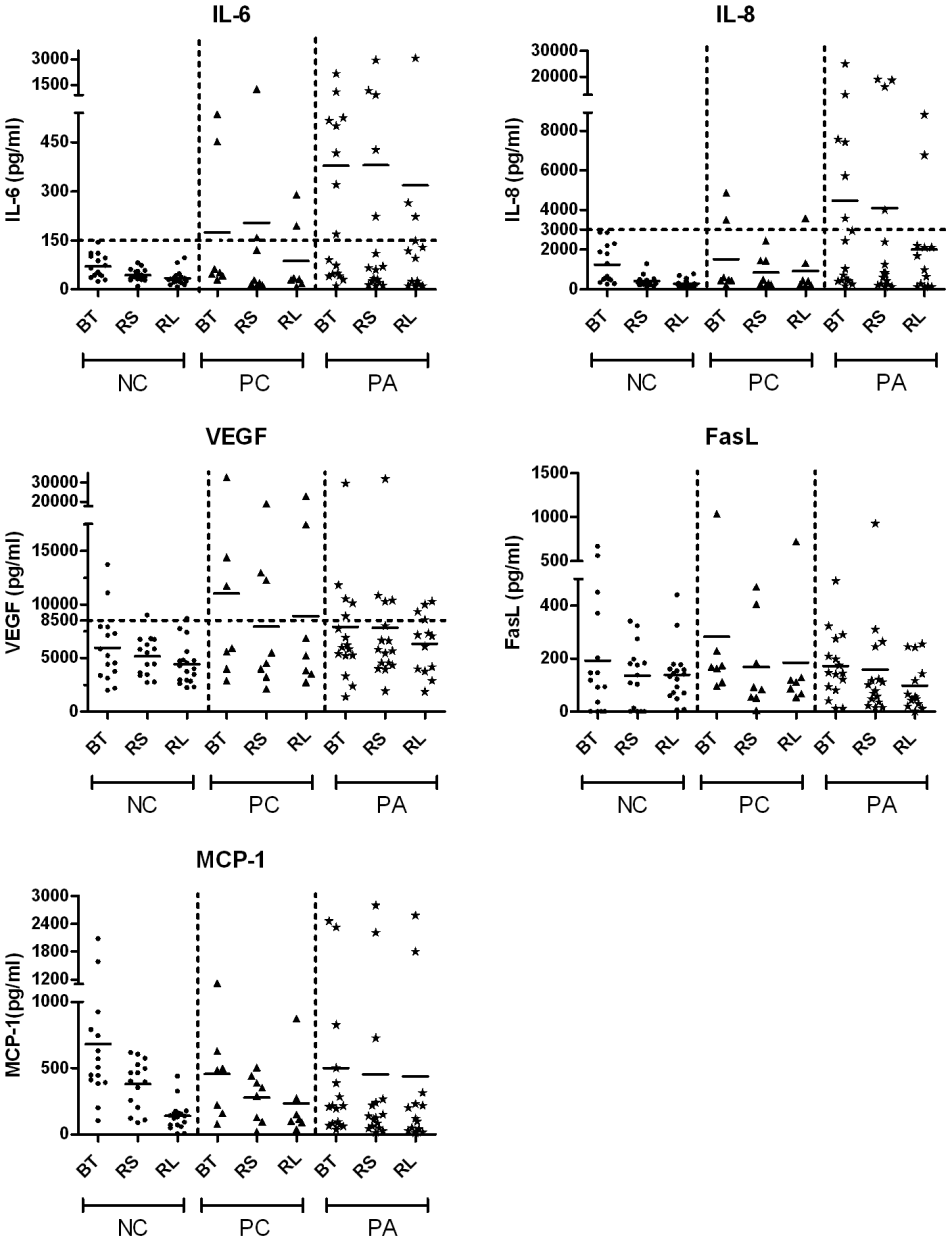
Overview of cytokine profiles in normal control (NC), patient control (PC) and patient affected (PA) groups. Basal tears (BT), Reflex tears collected from the inferior fornix in Sitting position (RS) and Reflex tears collected from the superior fornix in the Lying position (RL) were compared for the different study groups. No significant differences were observed (p>0.05).

Tear cytokine in BT, RS and RL samples were compared for the three study groups namely: PA, PC and NC. Corneal samples were not included since these were diluted during collection unlike BT, RS and RL. No significant differences were seen between the three groups for the 5 analytes measured (Figure 5). However, taking a closer look at IL-6 and IL-8, there seemed to be a trend for higher expression of the respective cytokines in the PA eyes compared to the controls. By demarcating a threshold at 150 pg/ml for IL-6; 8 of the 15 PA eyes in the BT group showed levels above threshold compared to none of the NC eyes in the BT group (*p* = 0.0019). Similarly, by setting a threshold at 3000 pg/ml for the cytokine IL-8, we noticed 7 eyes in the BT of PA eyes that were above the threshold compared to none of the BT of NC eyes. Differences were less pronounced for VEGF, MCP-1 and sFasL.

### Cytokine Profile in Corneal Tear Samples

**Figure 6:**
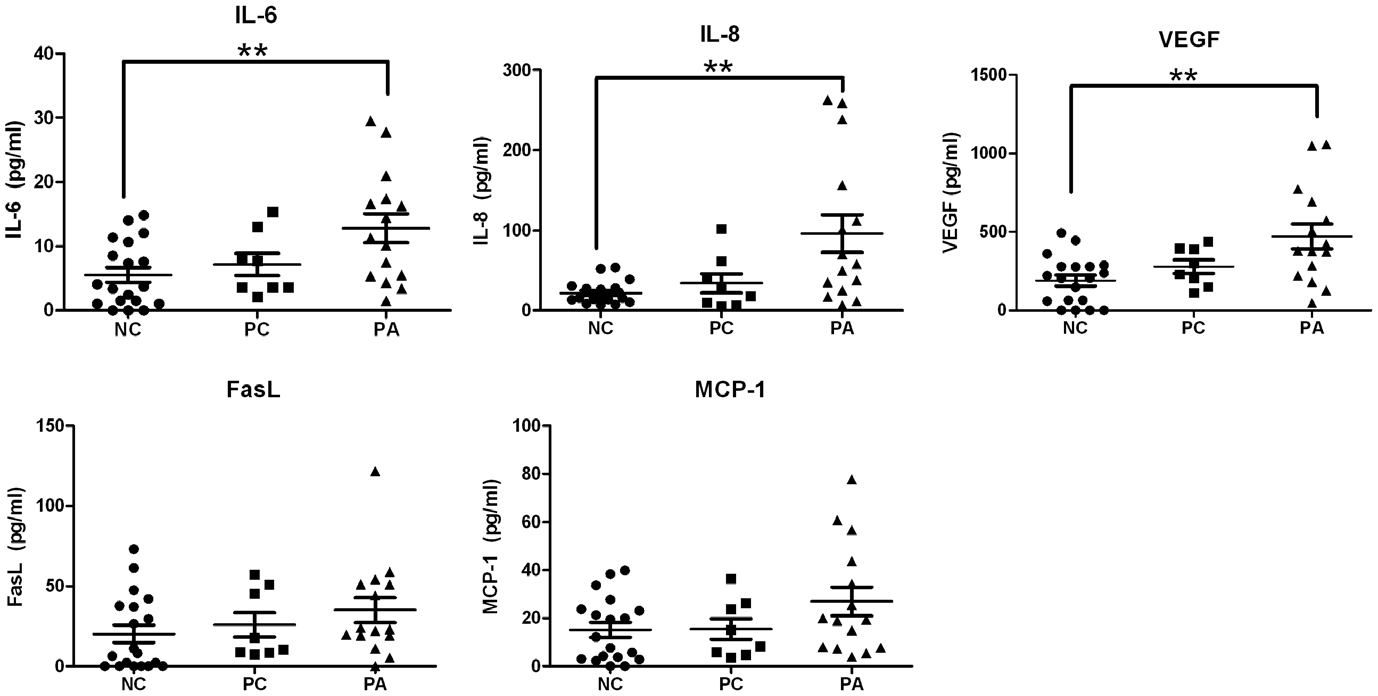
Cytokine concentrations in corneal epithelial secretions from normal and vascularized eyes. Cytokine levels of tears collected with a cornea bath (100× diluted) were measured for the Normal Control group (NC, n = 20), Patient’s Control group (PC, n = 8) and Patients Affected group (PA, n = 16). Horizontal lines represent mean Concentrations with SEM. Differences are significant if **p<0.001.

**Figure 7:**
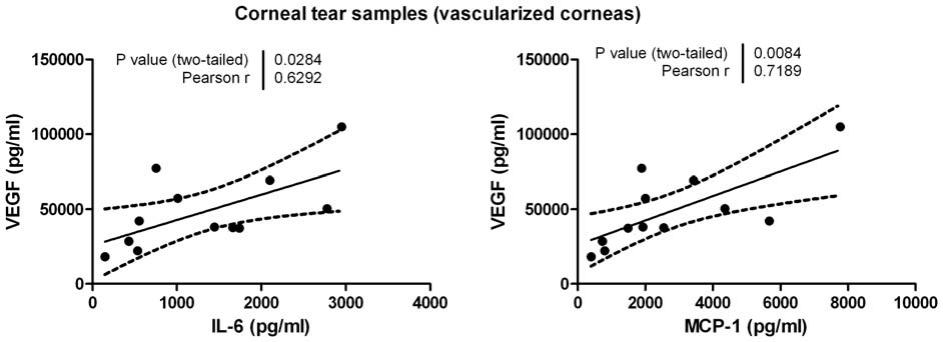
Correlations between the concentrations VEGF and IL-6 and VEGF and MCP-1 in corneal tear samples of vascularized corneas. Pearson correlations are significant if *p*<0.05.

Corneal tear samples collected using the corneal bath showed significantly higher levels of IL-6, IL-8 and VEGF in the vascularized group compared to the NC group (Figure 6). Since there was no significant difference between the PC and NC groups (FDR-*p*>0.05) these groups were combined in order to increase the cohort and termed “all control”. PA versus all control maintained significance for IL-6, IL-8 and VEGF despite performing corrections for multiple testing (FDR-*p*<0.05). Interestingly, we could detect in corneal tear samples of vascularized corneas a positive correlation between the concentration of VEGF and the cytokine IL-6 (*p* = 0.028) and VEGF and the cytokine MCP-1 (*p* = 0.008; Figure 7).

### Relationship between Cytokine Concentrations in Tears and Area of Vascularization

**Figure 8:**
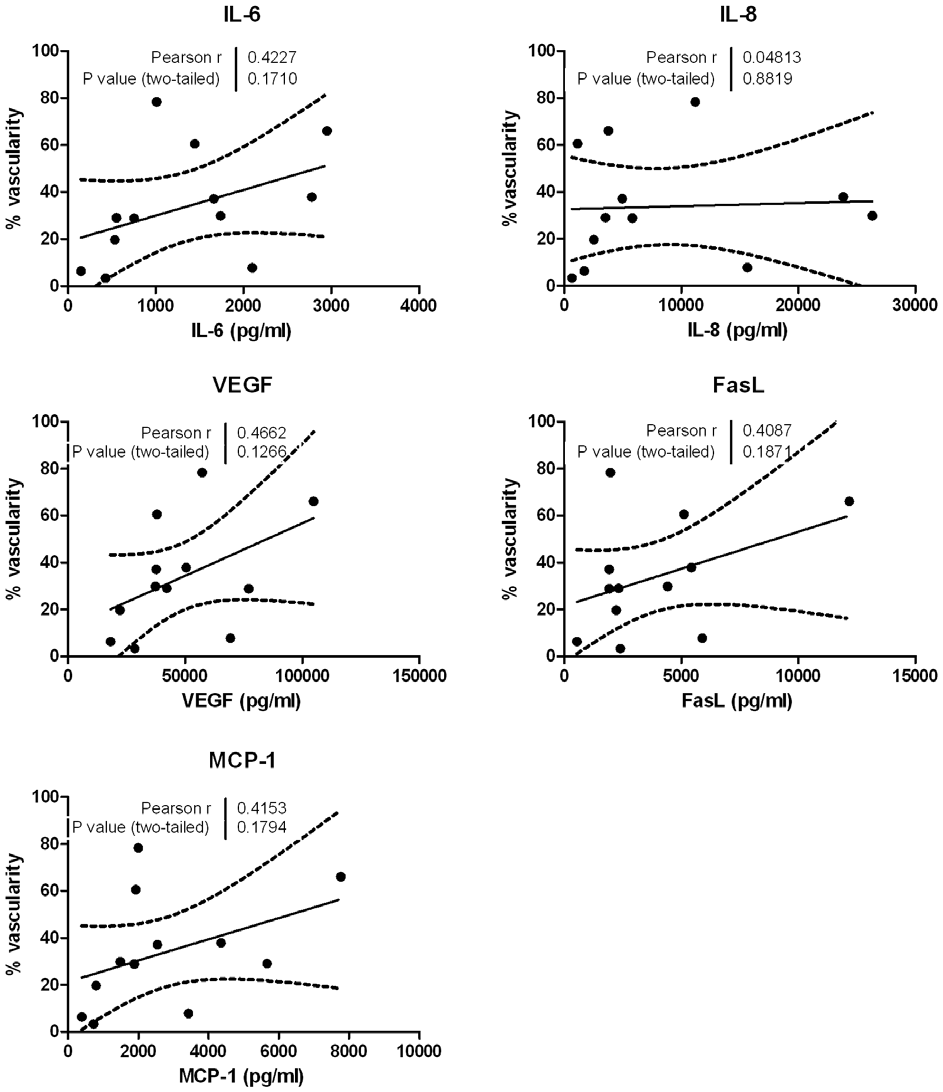
Correlations of % vascularization and levels of cytokines in corneal tear samples collected using a corneal bath. The % area vascularization was calculated as the sum of small, medium and large vessels and compared to the cytokine levels in corneal epithelial secretions. For these experiments, 12 cornea’s of 9 patients were included. Correlations are statistically significant if p<0.05 and the dotted lines indicate the 95% confidence intervals.

The overall % area of vascularization of the cornea of each affected eye (n = 12; PA group, n = 9) was calculated by the image processing software as the sum of the % area of Small Vessels (SV), % area of Medium Vessels (MV) and the % area of Large Vessels (LV) (Figure 8). The total % vascularization was put in relation with the concentration of cytokines detected in the corneal epithelial secretions collected using the corneal bath. We could not detect any significant correlations between % vascularization and IL-6, IL-8, VEGF, FasL or MCP-1 (Figure 8).

## Discussion

Tears are secreted from the main and accessory lacrimal gland and pass laterally across the ocular surface. After gas exchange between the air and the epithelium, the tears drain into the canaliculi through the puncta. Tears contain a wide array of cytokines and growth factors, secreted by the lacrimal gland as well as some cytokines that are locally produced and diffuse into the tear film from the corneal and conjunctival epithelia. Since cytokines are freely diffusible, they can also leak into the tear film from the conjunctival blood vessels.

In this study we demonstrate that there are pro-angiogenic cytokines present in tears of normal eyes which are at significantly higher levels than those found in the serum. This is the first study providing a standardized comparison between the angiogenic cytokine composition of human tears and serum. These large variations in cytokine tear and serum levels provide compelling evidence for their localized corneal production and secretion into the tear film. Furthermore, analysis of the tears taken from different collection points revealed on average highest concentrations in BT followed by RS and RL. Since reflex tears have a higher flow rate than basal tears our results are not surprising. Similar observations were made when comparing basal and reflex tears for IL-8 levels by Sonoda et al. [9]. The differences between RS and RL (Figure 5) for the different cytokines, though not marked, displayed a trend towards lower cytokine levels in RL compared to RS. Again this outcome probably relates to the fact that the RL tears are picked up as soon as they leave the lacrimal gland whereas the RS tears spend more time on the ocular surface prior to collection allowing time for diffusion of cytokines from the epithelia into the tear film.

After analyzing the tear samples taken from different collection points, no significant differences were seen in the NC, PC and PA groups when comparing the basal or reflex tears (Figure 5). Interestingly, when the corneal epithelial secretions from the same groups were compared, significant differences were observed between the patients’ affected eyes and the normal controls (Figure 6). These data together suggest that due to the localized corneal cytokine production, the best method for collecting tear samples of patients with corneal neovascularization is to collect a fixed dilution of the localized corneal tears using a silicone corneal bath.

MCP-1 levels in vascularized corneas were higher than in the control group although this difference did not reach significance. Damage to corneal epithelial basal cells prompts their release of MCP-1 initiating the macrophage chemotactic response [10]. The activated macrophages release VEGF [11], [12] which induces the recruitment of activated endothelial cells from the surrounding blood vessels. The endothelial cells complete the vicious circle by subsequently up-regulating the release of MCP-1 [13]. Interestingly, we observed a strong positive correlation between MCP-1 and VEGF (ure 7, *p* = 0.008) proving our initial hypothesis that implicated MCP-1 in the VEGF pathway. It is also plausible that MCP-1 and VEGF are both downstream of the same angiogenic signaling pathway (IL-1α, -1β) which may also explain the strong correlation. Further research is warranted in order to prove this. The nearly 2 fold increase in the mean concentration of MCP-1 in vascularized corneas suggests it is an important factor playing a role in angiogenesis. In this context, Parenti et al [12] demonstrated a significant increase in *ex vivo* vascular smooth muscle cell migration when stimulated with as little as 10 pg/ml of MCP-1, whereas in our study we observed increase in mean concentrations from 379 pg/ml (in PC group) to 501 pg/ml (in PA group). Parenti et al. also observed that the potency of MCP-1 dependant cell migration increased considerably in areas of hypoxia, which leads us to believe that even though there wasn’t a significant increase in MCP-1 concentration in vascularized corneas, it could be highly relevant.

The VEGF levels in the corneal tear samples of the vascular corneas were 2.5 times higher than in those of the control subjects. This confirmed our former hypothesis implicating VEGF as one of the main mediators in corneal angiogenesis.

The higher levels of IL-6 (1.3 fold increase), IL-8 (1.6 fold increase) and VEGF (1.5 fold increase) observed in the contralateral normal eyes of the vascular group (PC group, Figure 5) compared to the NC group, may have some clinical relevance. This could indicate that both eyes respond to stimulus as a single organ with an undesirable increase in pro-angiogenic analytes in the tear film of the normal contralateral eye. Future minor insults in this sympathetic eye could therefore potentially lead to disastrous consequences due to an augmented response. These deductions are premature but do necessitate further evaluation in animal models. A limitation of this study is the small sample size and therefore a slightly larger scale study may be required to valorize the results obtained.

No significant correlations were observed for the cytokine levels and degree of corneal vascularization. It is possible that this is because not all the corneas were in the same stage of active vascularization. Perhaps using animal models of corneal neovascularization could help attain a more homogenous population and significant correlations with angiogenic cytokine levels.

In summary, human tears provide a dynamic reflection of soluble factors present at the ocular surface. During the course of this study, we compared different tear collection points and were able to provide evidence for the localized production and secretion of pro-angiogenic cytokines into the tear film, necessitating the importance of site and method of tear collection. Moreover we performed a comparative analysis between corneal tears from patients with vascularized corneas and those from a normal control group showing statistically significant increases in IL-6, IL-8 and VEGF. The results are encouraging since this is the first study of its kind which has succeeded in highlighting significantly higher levels of pro-angiogenic cytokines in tears from patients with neovascular corneas. Inter-cytokine dependence was studied and we found strong correlation of IL-6 and MCP-1 with the highly angiogenic VEGF in human tear fluid. These results not only underscore the foreseeable use of antiangiogenic drugs such as the monoclonal antibody against VEGF for the treatment of corneal neovascularization [8], but substantiates a combined, multi-drug approach using anti-IL-6, anti-MCP-1 or anti-IL-8 in addition. Finally we were also able to identify trends for modestly increased levels of angiogenic analytes in the contralateral ‘normal’ eyes of patients with unilateral corneal neovascularization, indicating the role of these biomarkers in diagnosis of sympathetic ophthalmia. This non-traumatic technique for analyzing local inflammatory mediators in tears has far reaching implications in ocular diagnostics and warrants further investigation.
